# Environmental levels of avian antigen are relevant to the progression of chronic hypersensitivity pneumonitis during antigen avoidance

**DOI:** 10.1002/iid3.202

**Published:** 2017-11-22

**Authors:** Manabu Sema, Yasunari Miyazaki, Toshiharu Tsutsui, Makoto Tomita, Yoshinobu Eishi, Naohiko Inase

**Affiliations:** ^1^ Department of Respiratory Medicine Tokyo Medical and Dental University 1‐5‐45 Yushima Bunkyo‐ku Tokyo 113‐8519 Japan; ^2^ Department of Clinical Research Center Tokyo Medical and Dental University 1‐5‐45 Yushima Bunkyo‐ku Tokyo 113‐8519 Japan; ^3^ Department of Human Pathology Tokyo Medical and Dental University 1‐5‐45 Yushima Bunkyo‐ku Tokyo 113‐8519 Japan

**Keywords:** Antigen avoidance, avian antigen, chronic hypersensitivity pneumonitis

## Abstract

**Background:**

In chronic hypersensitivity pneumonitis (chronic HP), antigen avoidance is critical for disease management; however, complete avoidance is difficult because of unrecognized exposure to antigens. Recently, we revealed that the amount of avian antigen (AAA) in household dust at the time of diagnosis predicted the progression of chronic bird‐related HP. The purpose of this study is to evaluate the relationship between the prognosis of chronic bird‐related HP and the AAA that remained in the environment during antigen avoidance.

**Methods:**

First, we measured the AAA in household dust of 28 consecutive patients (22 with chronic bird‐related HP and 6 with acute bird‐related HP) and 12 healthy volunteers. Second, we measured the AAA and collected questionnaires on the environmental conditions of the homes of 53 patients with various lung diseases, including bird‐related HP, to investigate the environmental parameters related to a higher AAA. Finally, we prospectively recruited 14 consecutive patients with chronic bird‐related HP, measured the AAA periodically, and collected clinical data.

**Results:**

The AAA was higher in patients with chronic bird‐related HP at the time of diagnosis compared to healthy volunteers and was highest in patients with acute bird‐related HP. Logistic regression analysis showed that birds frequenting a residence was the only significant factor for a higher AAA (odds ratio, 5.686; 95%CI, 1.263–25.59; *P* = 0.024). There was a correlation between the mean AAA and decline of vital capacity for 1 year (*r* = −0.55; 95%CI −0.84 to −0.01; *P* = 0.043).

**Conclusion:**

Measurements of the AAA after diagnosis predict the progression of chronic bird‐related HP. Avian antigen can exist in the indoor environment regardless of antigen avoidance. The presence of avian antigen in the indoor environment can be attributed to wild birds found outdoors.

## Introduction

Hypersensitivity pneumonitis (HP) is an interstitial lung disease that is caused by an allergic reaction following inhalation of fungi, bacteria, acid‐fast organisms, animal proteins, metallic dust, and so on 1. HP is classified as acute or chronic 2, 3. According to a national survey in Japan, avian antigen is the cause of chronic HP in 61% of cases 4.

Antigen avoidance is critical in the management of chronic HP 5. Bird‐related HP is caused by not only direct exposure (breeding birds) but also unrecognized exposure (feather products, wild birds, and breeding of birds by neighbors) according to previous reports 6, 7, 8, 9, 10, 11, 12. Unrecognized exposure is unlikely to be avoided. Therefore, it is critical to measure the amount of avian antigen (AAA) in the environment in patients with HP.

In a few previous studies, measurement of the AAA by competitive or antigen‐capture ELISA was reported 13, 14, 15, but these studies were unable to detect low concentrations of AAA and did not examined the relationship between the AAA and clinical course of chronic bird‐related HP. Recently, we developed a sandwich ELISA assay to measure the AAA, and reported that the AAA present in household dust correlated with the prognosis of chronic bird‐related HP 16. However, it is unknown how the AAA changes with time after diagnosis during antigen avoidance, and whether the AAA relates to the clinical course and prognosis of chronic bird‐related HP. Chronic HP has been assumed to progress as a result of either persistent exposure to very small amounts of antigen or unrecognized exposure even after optimal antigen removal from the environment 13, 17. Therefore, it is important to examine the AAA after diagnosis and during antigen avoidance.

We planned step‐by‐step studies. First, to examine the background data on the AAA in chronic bird‐related HP, we compared the AAA at the time of diagnosis in patients with chronic bird‐related HP and healthy volunteers (Retrospective Dust Study). Second, using a questionnaire, we investigated the relationship between the AAA and environmental conditions (Questionnaire Dust Study). Finally, we periodically measured the AAA in the environment of patients with chronic bird‐related HP after diagnosis and during antigen avoidance (Prospective Dust Study).

## Materials and Methods

### Informed consent

Informed consent was obtained from all subjects. Information about this study was disclosed to the study participants through the institutional review board and was approved by the ethical research committee at the Tokyo Medical and Dental University (approval number: 1578, M2016‐189).

### Measurement of the AAA

The sampling method for household dust collection was based on the protocol of Tsutsui et al. with slight modifications 16. Household dust was obtained from the living rooms and bedrooms where each patient spent the most time via a domestic vacuum cleaner with a 40‐µm pore‐size sampling filter (Dustream Collector, Indoor Biotechnologies, Inc., Charlottesville, VA).

Approximately 100 mg of dust from each sample was added to 4 ml of PBS with Tween 20 and was shaken for 2 h at room temperature. After centrifugation for 5 min at 500 × g, the supernatant solution was filtered through a 40 µm filter (Cell Strainer, BD Biosciences, San Jose, CA), then filtered through a 0.45 µm syringe filter (Stertile Acrodisc® Syringe Filters with Supor® Membrane, PALL Life Sciences, Ann Arbor, MI). The dust extract solutions were stored frozen at −20°C until analysis.

Monoclonal and polyclonal antibodies against pigeon dropping extract were generated according to the methods of previous studies 15, 16. A sandwich ELISA with monoclonal and polyclonal antibodies was used to measure the AAA as described previously 16. To increase the sensitivity of avian antigen detection, we used the ELAST amplification system (ELAST®, PerkinElmer Life Sciences, Inc., Waltham, MA) per the manufacturer's instructions. The AAA is expressed in micrograms of pigeon dropping extract per 1 gram of dust.

### Retrospective dust study

#### Subjects

We undertook a retrospective review of the medical records of inpatients and outpatients who were diagnosed with acute or chronic bird‐related HP from April 2011 to October 2016 at the Tokyo and Dental University Medical Hospital, a 753‐bed tertiary care hospital in a major urban center in Tokyo. The diagnosis of acute bird‐related HP was based on acute symptoms, a history of contacting birds or bird‐related products, ground glass opacity on a chest CT, and high titre of an antibody to a bird. The diagnosis of chronic bird‐related HP was based on chronic symptoms, radiological images of chronic interstitial pneumonitis (reticular shadow, honeycombing), and a positive result of the bird‐antigen inhalation provocation test (IPT). The bird‐antigen IPT was performed according to the method described in a previous study 18. The criteria for a positive result depended on the IPT prediction score (IPT‐PS), as reported in a previous study 19. We excluded patients for whom household dust was not examined. In addition, as a control group, we selected healthy volunteers from the attending staff of our department.

#### Sampling of household dust

We collected dust from the households of patients with acute bird‐related HP or chronic bird‐related HP from April 2011 to October 2016 at the time of diagnosis as well as dust from the households of healthy volunteers from August 2016 to October 2016. We measured the concentration of avian antigen in household dust as described in the “Measurement of the AAA” section.

### Questionnaire dust study

#### Subjects

We randomly recruited outpatients and inpatients with various lung diseases (including interstitial pneumonitis, lung cancer, bronchial asthma, etc.) who visited our department from April 2016 to May 2017. We excluded patients from whom we could not obtain consent or patients who raised a bird indoors or outdoors.

#### Questionnaire and classification of patients

We asked the recruited patients to collect household dust by themselves via a domestic vacuum cleaner with a sampling filter as described in the “Measurement of the AAA” section and to complete a questionnaire on the indoor and outdoor environmental conditions. We sent the sampling filter and questionnaire to the households of patients by mail and asked them to return the samples of household dust and completed questionnaire. We measured the AAA and determined the relationship between the AAA and environmental characteristics. We classified the recruited patients into the high or low‐level exposure group with a cut‐off value of the AAA (0.74 µ/g of dust) that was reported to predict the prognosis of chronic bird‐related HP in the previous study 16.

### Prospective dust study

#### Subjects

We prospectively recruited patients with chronic bird‐related HP who were diagnosed on the basis of bird‐antigen IPT in our hospital after July 2006 and who visited our hospital from April 2011 to August 2015. We excluded patients who could not undergo a pulmonary function test because of illness, did not provide consent at the time of recruitment, or who changed hospitals before they underwent a year of follow‐up visits.

#### Clinical tests and household dust

Each patient underwent clinical laboratory testing and spirometry testing and gathered household dust every 3 months for 1 year from the initial recruitment. The clinical and environmental characteristics at the time of recruitment were evaluated for each group.

#### Classification of patients

We classified patients into the high exposure group, in which the mean AAA for 1 year was higher than 0.74 µg/g of dust, and the low exposure group, in which the mean AAA for 1 year was lower than 0.74 µg/g of dust. We compared the annual decline of VC and FVC between the two groups.

### Statistical analysis

Statistical analyses were performed with PRISM (GraphPad, San Diego, CA) and SPSS statistics version 17.0 (IBM Corp., Armonk, NY). In all studies, continuous and categorical variables between two groups were compared with the Mann–Whitney U test and Fisher's exact probability test, respectively. Continuous and categorical variables were compared among the three groups via the Kruskal–Wallis test and Fisher's exact probability test, respectively. In the Questionnaire Dust Study, factors associated with a high AAA were evaluated by logistic regression analysis. In the Prospective Dust Study, Spearman rank correlation was used to determine the relationship between the mean AAA collected for 12 months and annual decline of %VC from baseline. All statistical comparisons were two‐sided, and *P*‐values less than 0.05 were considered statistically significant.

## Results

### Retrospective dust study

#### Patient selection

**Figure 1:**
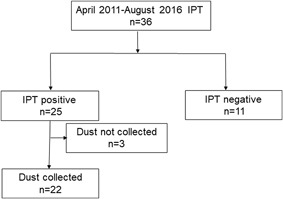
Diagram outlining the patient selection of chronic bird‐related HP in the retrospective dust study. IPT, inhalation provocation test.

Patient selection in the Retrospective Dust Study is illustrated in Figure 1. From April 2011 to October 2016, six patients were diagnosed with acute bird‐related HP, and dust was collected from the households of all patients. Bird‐antigen IPT was performed in 36 patients with chronic interstitial pneumonitis and possible bird‐related HP, 25 of whom had a positive IPT result and were diagnosed with chronic bird‐related HP. The household dust of 22 consecutive patients with chronic bird‐related HP was examined at the time of diagnosis. The household dust of three patients was not collected because patients changed hospitals after diagnosis or consent was not obtained. The household dust of 12 healthy volunteers was also examined as a control group.

#### Characteristics of patients

The characteristics of patients in the Retrospective Dust Study are shown in Table 1. The healthy volunteers were younger than patients in other groups. All patients with acute bird‐related HP were women, and almost all of them raised a bird. On the other hand, only one patient with chronic bird‐related HP was currently raising a bird. Healthy volunteers were more likely to have bird related products than other groups (feather duvet: 75%, *P* = 0.08; down jacket: 75%, *P* = 0.0041).

**Table 1:** Characteristics in the retrospective dust study^a^

Characteristics	Acute bird‐related HP (*n* = 6)	Chronic bird‐related HP (*n* = 22)	Healthy volunteer (*n* = 12)	*P* value
Age (year)	57 (45–63)	68 (64–72)	33 (31–35)	<0.0001
Sex, Male, *n* (%)	0 (0)	11 (50)	9 (75)	0.011
Smoking (pack‐years)	0 (0–1)	0 (0–23)	0 (0–0)	0.10
VC^b^ (%)	84 (75–95)	69 (62–76)		0.05
KL‐6 (U/ml)^c^	1866 (976–5241)	1257 (799–1946)		0.25
SP‐D (ng/ml)^d^	333 (113–670)	245 (154–481)		0.97
Bird raising^e^, *n* (%)	5 (83)	1 (4.5)	0 (0)	<0.0001
Possession of duvet feather, *n* (%)	2 (33)	8 (36)	9 (75)	0.08
Possession of down jacket, *n* (%)	2 (33)	4 (18)	9 (75)	0.0041
Possession of poultry manure, *n* (%)	1 (16)	2 (9)	0 (0)	0.25
Possession of stuffed bird, *n* (%)	1 (16)	1 (4.5)	0 (0)	0.36
Location of residence^f^, *n* (%)				
In the city	3 (50)	10 (45)	10 (83)	0.10
In the suburbs	3 (50)	12 (55)	2 (17)	

^a^ The extent of each continuous variable of characteristics is presented as the median (25th and 75th percentiles).

^b^ VC, vital capacity.

^c^ KL‐6, sialylated carbohydrate antigen KL‐6.

^d^ SP‐D, surfactant protein‐D.

^e^ Burd raising represents current raising at the time of diagnosis, not historic contact with a bird.

^f^ The city was defined, as noted, as the central city in Kanto on Japanese national census: Tokyo borough, Yokohama City, Kawasaki City, Chiba City, Saitama City. The suburbs were defined as areas other than above areas.

#### Comparison of AAA among groups

**Figure 2:**
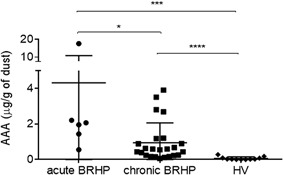
Comparison of AAA between groups in the Retrospective Dust Study. AAA, amount of avian antigen; BRHP, bird‐related hypersensitivity pneumonitis; IIPs, HV, healthy volunteer. *P <0.05, ***P <0.001, ****P <0.0001.

In the Retrospective Dust Study, the AAA in acute bird‐related HP patients was higher than in chronic bird‐related HP patients (4.30 ± 6.54 µg/g of dust vs. 0.94 ± 1.10 µg/g of dust; *P* = 0.035). The AAA in chronic bird‐related HP patients was higher than the AAA in healthy volunteers (0.94 ± 1.10 µg/g of dust vs. 0.06 ± 0.08 µg/g of dust; *P *< 0.0001) (Fig. 2).

### Questionnaire dust study

#### Patient selection

In the Questionnaire Dust Study, both the questionnaire and house dust samples were collected from 53 patients, including 16 patients with chronic bird‐related HP, 23 patients with idiopathic interstitial pneumonitis (IIPs), 7 patients with home‐related HP, and 7 patients with other diseases (microscopic polyangiitis, sleep apnea syndrome, bronchial asthma, bacterial pneumonia, pleural mesothelioma, and sarcoidosis).

#### Relationship between the environment and AAA

In the Questionnaire Dust Study, we compared environmental characteristics between the high‐level exposure group, in which the AAA was higher than 0.74 µg/g of dust, and the low‐level exposure group, in which the AAA was lower than 0.74 µg/g of dust (Table 2). In the high‐level exposure group, more patients reported on their questionnaire that wild birds frequently visited their garden or veranda and that bird droppings were often found in their garden or veranda. As shown in Table 3, logistic regression analysis of the high‐level exposure of avian antigen was performed with the following variables: the location of the residence, floor number of dust, frequency of birds visiting the garden or veranda, frequency of bird droppings in the garden or veranda, and existence of a field or forest around the residence. These five items were included in the logistic regression analysis because they had *P* values of almost less than 0.20. In univariate analysis, the frequency of birds visiting the garden or veranda and frequency of bird droppings in the garden or veranda were identified. In the multivariable models, the location of the residence, the floor number, the frequency of birds visiting the garden or veranda and existence of a field or forest around the residence were included. The frequency of bird droppings in the garden or veranda was excluded in the multivariable models because of a significant correlation with the frequency of birds visiting the garden or veranda. The frequency of bird droppings in the garden or veranda was the only significant variable (odds ratio 5.686; 95%CI 1.263–25.59; *P* = 0.024) (Table 3).

**Table 2:** Environmental characteristics in the Questionnaire Dust Study^a^

Characteristics	High‐level exposure group (*n* = 13)	Low‐level exposure group (*n* = 40)	*P* value	
Location of residence (city/suburbs)^b^	5/8	24/16	0.21	
Type of house construction (detached/complex)	10/3	23/17	0.32	
Age of house (years)	23 (20–39)	22 (15–33)	0.48	
Floor number	2.0 (1.0–2.0)	2.0 (1.0–4.0)	0.21	
Possession of duvet feather, *n* (%)	3 (23)	9 (24)	1.00	
Possession of down jacket, *n* (%)	5 (38)	9 (24)	0.29	
Possession of avian manure, *n* (%)	2 (15)	2 (4.8)	0.24	
Possession of stuffed bird, *n* (%)	0 (0)	0 (0)	1.00	
Frequent birds coming to garden or veranda^c^, *n* (%)	7 (53)	5 (12)	0.0047	
Frequent bird droppings in garden or veranda^d^, *n* (%)	5 (38)	2 (4.8)	0.007	
Existence of field or forest around the residence, *n* (%)	8 (61)	15 (36)	0.19	
Times of cleaning the room (times a week)	4.0 (2.5–7.0)	3.0 (1.0–5.0)	0.24	
Usage of air cleaner in the room (hours a day)	0.0 (0.0–12.5)	0.0 (0.0–24)	0.66	

^a^ The extent of each continuous variable of characteristics is presented as the median (25th and 75th percentiles).

^b^ The city was defined, as noted, as the central city in Kanto on Japanese national census: Tokyo borough, Yokohama City, Kawasaki City, Chiba City, Saitama City. The suburbs were defined as areas other than the above areas.

^c^ Questions on frequency of birds coming to the garden or balcony; frequently or rarely or not at all. Analyzed by binary manner; frequent or not frequent.

^d^ Questions on frequency of bird droppings in the garden of balcony; frequently or rarely or not at all. Analyzed by binary manner; frequent or not frequent.

**Table 3:** Logistic regression of factors associated with high‐level exposure of avian antigen in the Questionnaire Dust Study

Variables	Unadjusted odds ratio	Unadjusted 95% confidence interval	*P* Value	Adjusted odds ratio^a^	Adjusted 95% confidence interval	*P* Value
Location of residence (city/suburbs)^b^	2.400	0.665–8.666	0.18	1.136	0.237–5.448	0.87
Floor number	0.564	0.264–1.205	0.13	0.721	0.317–1.638	0.43
Frequent birds coming to garden or veranda^c^	8.167	1.939–34.39	0.004	5.686	1.263–25.59	0.02
Frequent birds dropping in garden or veranda^d^	11.87	1.947–72.44	0.007	–	–	–
Existence of field or forest around the residence	2.667	0.736–9.665	0.13	1.695	0.372–7.731	0.49

^a^ Odds ratio adjusted for the location of residence, the floor number, the frequent birds coming to the garden, existence of field or forest around the residence.

^b^ Data are analyzed as city:0; suburbs:1.

^c^ Data are analyzed as rare or no birds coming to garden:0; frequent birds coming to garden:1.

^d^ Data are analyzed as rare or no birds dropping in garden:0; frequent birds dropping in garden:1.

### Prospective dust study

#### Patient recruitment

**Figure 3:**
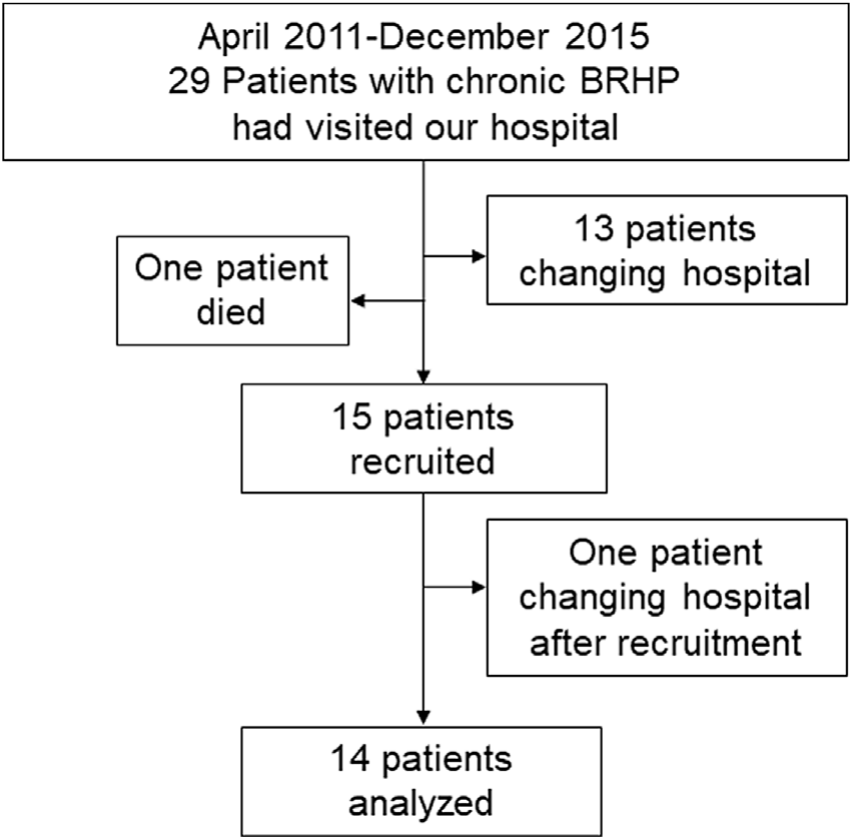
Diagram outlining the patient recruitment in the Prospective Dust Study. BRHP, bird‐related hypersensitivity pneumonitis.

The patient recruitment process for the Prospective Dust Study is shown in Figure 3. From April 2011 to August 2015, a total of 29 patients with chronic bird‐related HP visited our hospital after diagnosis based on the results of their IPT. Of the 29 patients, 13 were excluded because they changed hospitals and one was excluded because they died before recruitment. This resulted in the recruitment of 15 chronic bird‐related HP patients. One patient changed hospitals after recruitment and was unable to be followed for a full year. Thus, 14 patients were included in the analysis of this study.

#### Patient characteristics

In the Prospective Dust Study, of 14 patients who participated in the study, 4 were classified into the high‐level exposure group and 10 were classified into the low‐level exposure group. The clinical and environmental characteristics of the two groups are shown in Table 4. The follow‐up period after diagnosis of chronic bird‐related HP was 1 year or more. Because of medical guidance to avoid avian antigen in the patient's environment, no patients used bird‐related products except one patient in the low exposure group who used a feather duvet. Significantly more patients in the high‐level exposure group resided in the suburbs than in the low‐level exposure group.

**Table 4:** Characteristics at the time of recruitment in the high and low‐level exposure group in the Prospective Dust Study^a^

Characteristics	High‐level exposure group (*n* = 4)	Low‐level exposure group (*n* = 10)	*P* value
Age (year)	69 (61–75)	66 (61–70)	0.55
Sex, Male, *n* (%)	4 (100)	3 (30)	0.069
Smoking (pack‐years)	11 (0–29)	0 (0–13)	0.52
Period after diagnosis of CBRHP (months)	14 (2.7–29)	25 (11–46)	0.43
VC^b^ (%)	69.0 (58.5–74.9)	75.9 (67.0–100)	0.14
KL‐6^c^ (U/ml)	878 (628–1544)	1038 (548–2865)	0.73
SP‐D^d^ (ng/ml)	355 (324–541)	149 (71.3–322)	0.072
Treatment, *n* (%)			
No treatment	3 (75)	3 (30)	0.24
Corticosteroid	1 (25)	7 (70)	
Bird nursing, *n* (%)	0 (0)	0 (0)	1.00
Possession of down jacket, *n* (%)	0 (0)	0 (0)	1.00
Possession of duvet feather, *n* (%)	0 (0)	1 (10)	1.00
Possession of poultry manure, *n* (%)	0 (0)	0 (0)	1.00
Possession of stuffed bird, *n* (%)	0 (0)	0 (0)	1.00
Location of residence^e^, *n* (%)			
In the city	0 (0)	8 (80)	0.015
In the suburbs	4 (100)	2 (20)	

^a^ The extent of each continuous variable of characteristics is presented as the median (25th and 75th percentiles).

^b^ VC, vital capacity.

^c^ KL‐6, sialylated carbohydrate antigen KL‐6.

^d^ SP‐D, surfactant protein‐D.

^e^ The city was defined as the central city in Kanto on Japanese national census: Tokyo borough, Yokohama City, Kawasaki City, Chiba City, Saitama City. The suburbs were defined as areas other than above areas.

#### AAA and pulmonary function tests

**Figure 4:**
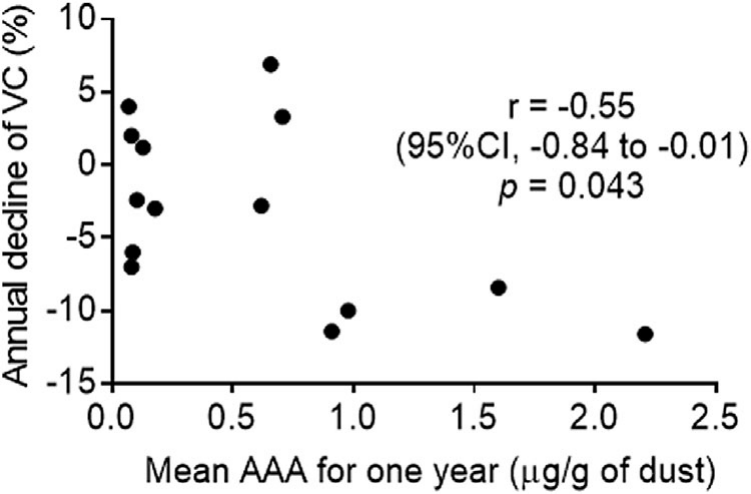
Relation between mean AAA for 1 year and annual decline of VC% in the Prospective Dust Study. AAA, amount of avian antigen. VC, vital capacity.

**Figure 5:**
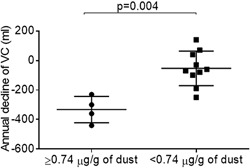
Comparison of the annual decline of VC (ml) between high and low‐level exposure groups in the Prospective Dust Study. VC, vital capacity.

**Figure 6:**
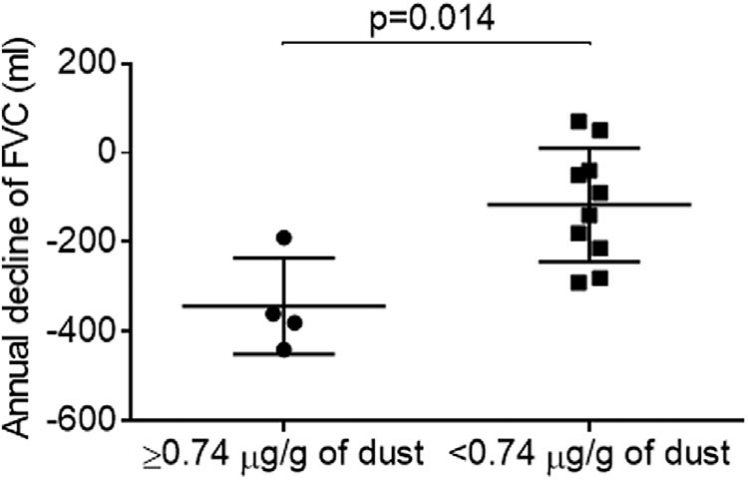
Comparison of the annual decline of FVC (ml) between high and low‐level exposure groups in the Prospective Dust Study. FVC, forced vital capacity.

As shown in Figure 4, in the Prospective Dust Study, a moderate correlation between the mean AAA over 12 months and annual decline of VC from baseline was detected (*r* = −0.55; 95%CI, −0.84 to −0.01; *P* = 0.043). The annual decline in VC from baseline in the high‐level exposure group was higher than in the low‐level exposure group (332 ± 89 ml vs. 53 ± 116 ml, *P* = 0.004) (Fig. 5), as was the annual decline in FVC (342 ± 107 ml vs. 116 ± 126 ml, *P* = 0.014) (Fig. 6).

## Discussion

These studies demonstrated that household dust avian antigen concentrations were higher among patients with HP than healthy controls, and patients with a higher AAA concentration had a worse trajectory of disease over 1 year of follow up than patients with a lower AAA concentration with existing disease.

The Retrospective Dust Study demonstrated that the AAA in the household dust of patients with acute bird‐related HP was much higher than in other groups. It was determined from the evidence that almost all patients with acute bird‐related HP raised birds in their homes. They had evidence of direct indoor residential exposure to avian antigen. The previous study also described an acute course in the patients with HP exposed to a large number of birds 20. It is possible that acute bird‐relate HP developed due to direct exposure to birds and a much higher AAA.

The Questionnaire Dust Study demonstrated that a high exposure of indoor avian antigen was associated with birds often visiting the house. We concluded that outdoor avian antigen entered the indoor environment, as suggested by a previous study 13. Another study demonstrated that exposure to birds from the surrounding environment was a significant predictor of IPT results and diagnosis of chronic bird‐related HP 21. Therefore, chronic bird‐related HP can develop from exposure to avian antigen that can come from outside the house.

The Prospective Dust Study demonstrated that in patients who were diagnosed with chronic bird‐related HP and avoided antigen exposure, the annual decline of VC was correlated with the mean AAA in household dust. Additionally, it was shown that the AAA can be high regardless of optimal indoor antigen avoidance (ex. eliminating feather duvets, down jackets, avian fertilizer, and stuffed birds). In a previous study, we reported that the AAA present at the time of diagnosis predicted chronic bird‐related HP 16. However, whether the AAA was lower during follow‐up and antigen avoidance and whether the AAA could still predict the progression of chronic bird‐related HP were not investigated. In the present study, it was shown that in the high‐level exposure group, in which avian antigen was present regardless of strict antigen avoidance, the decline of respiratory function was larger than in the low‐level exposure group. In the previous study, the AAA was reported to remain constant even several months after the cessation of nursing a bird 13; however, in most cases in the Prospective Dust Study, the use of bird‐derived products ceased for more than 1 year before the study. Given all of these findings and the results of the Questionnaire Dust Study, which showed that birds frequenting a residence was the only significant factor for a higher AAA, it is likely that the AAA is not relevant to indoor bird‐related products but rather to bird antigens outdoors.

Several limitations of this study should be acknowledged. First, the number of cases was small, especially in the high‐level exposure group in the Prospective Dust Study. It is challenging to diagnose chronic HP and evaluate patient eligibility because the avian inhalation test is limited due to the risk of disease exacerbation, even though the test is the gold standard for diagnosis. In addition, many patients were not available for follow‐up by our group because they had changed hospitals. Second, some patients had taken medication for interstitial pneumonitis (prednisolone was taken in all these cases). There was no significant difference in patients who had taken medication between the high and low‐level exposure groups; however, medication might influence the prognosis. Third, with respect to the Retrospective Dust Study, there was selection bias in the group of patients with chronic bird‐related HP diagnosed by the avian inhalation test because they were mostly selected by the presence of birds or bird droppings around their house. Fourth, in the Questionnaire Dust Study, there may be the differential recall bias between patients with chronic bird‐related HP and patients with other diseases. Patients who had been diagnosed chronic bird‐related HP might have been interested in birds as the cause of their illness, while patients with disease other than bird‐related HP might not have been. However, the bias should be minimal because we advised patients with IIPs that they should avoid the antigen, including avian proteins, because we cannot exclude the possibility of having HP. In addition, inclusion of patients with other respiratory diseases in the study may cause a bias. When we classified patients into the high‐level exposure group or low‐level exposure group in the three groups chronic bird‐related HP, IIPs or home‐related HP, and other diseases, there was no significant difference in the proportion of each category (*P* = 0.58, the Kruskal–Wallis test) (Supplemental Table S1). This finding indicates that there was not much bias among the groups.

## Conclusions

The present study demonstrated that avian antigen remained in the environment of patients regardless of the elimination of bird‐related products. We demonstrated that avian antigen derived from the outdoor environment predicted the progression of chronic bird‐related HP. Our study demonstrates the importance of measuring the amount of invisible avian antigen in the environment.

## Authors' Contributions

Yasunari Miyazaki, MD, PhD, had full access to all of the data in the study and takes responsibility for the integrity of the data and the accuracy of the data analysis. Manabu Sema, MD, and Yasunari Miyazaki, MD, PhD, contributed to study design, data collection, data analysis, statistical analysis, and manuscript preparation; Toshiharu Tsutsui, MD, PhD, contributed to study design, data collection. Makoto Tomita, PhD, contributed to statistical analysis. Yoshinobu Eishi, MD, PhD, contributed to production of monoclonal antibodies; Naohiko Inase, MD, PhD, contributed to study design and manuscript preparation.

## Conflicts of Interest

None of the authors have conflicts of interest to declare.

## Ethical Statement

All procedures used in this research were approved by the Ethical Committee of Tokyo Medical and Dental University. Written informed consent was obtained from the patients for publication of this Study.

## Supporting information

Additional supporting information may be found in the online version of this article at the publisher's web‐site.

**Table S1**. The classification of the patients with chronic bird‐related hypersensitivity pneumonitis, idiopathic interstitial pneumonias or home‐related HP, and other respiratory diseases into the high and low‐level exposure groups in the Questionnaire Dust Study.

## References

[1] Selman, M. , A. Pardo , and T. E. King, Jr 2012 Hypersensitivity pneumonitis: insights in diagnosis and pathobiology. Am. J. Respir. Crit. Care Med. 186:314–324. 22679012 10.1164/rccm.201203-0513CI

[2] Lacasse, Y. , M. Selman , U. Costabel , J. C. Dalphin , F. Morell , R. Erkinjuntti‐Pekkanen , N. L. Mueller , T. V. Colby , M. Schuyler , V. Jomphe , et al. 2009 Classification of hypersensitivity pneumonitis: a hypothesis. Int. Arch. Allergy Immunol. 149:161–166. 19127074 10.1159/000189200

[3] Yoshizawa, Y. , Y. Ohtani , H. Hayakawa , A. Sato , M. Suga , M. Ando . 1999 Chronic hypersensitivity pneumonitis in japan: a nationwide epidemiologic survey. J. Allergy Clin. Immunol. 103:315–320. 9949324 10.1016/s0091-6749(99)70507-5

[4] Okamoto, T. , Y. Miyazaki , T. Ogura , K. Chida , N. Kohno , S. Kohno , H. Taniguchi , S. Akagawa , Y. Mochizuki , K. Yamauchi , et al. 2013 Nationwide epidemiological survey of chronic hypersensitivity pneumonitis in Japan. Respir. Investig. 51:191–199. 10.1016/j.resinv.2013.03.004 23978646

[5] Miyazaki, Y. , T. Tsutsui , and N. Inase . 2016 Treatment and monitoring of hypersensitivity pneumonitis. Expert. Rev. Clin. Immunol. 12:953–962. 27117830 10.1080/1744666X.2016.1182426

[6] Greinert, U. , U. Lepp , and W. Becker . 2000 Bird Keeper's lung without bird keeping. Eur. J. Med. Res. 5:124. 10756167

[7] Saltoun, C. A. , K. E. Harris , T. L. Mathisen , and R. Patterson . 2000 Hypersensitivity pneumonitis resulting from community exposure to Canada goose droppings: when an external environmental antigen becomes an indoor environmental antigen. Ann. Allergy. Asthma. Immunol. 84:84–86. 10674570 10.1016/S1081-1206(10)62745-7

[8] Kokkarinen, J. , H. Tukiainen , A. Seppä , and E. O. Terho . 1994 Hypersensitivity pneumonitis due to native birds in a bird ringer. Chest 106:1269–1271. 7924510 10.1378/chest.106.4.1269

[9] Choy, A. C. , R. Patterson , A. H. Ray , and M. Roberts . 1995 Hypersensitivity pneumonitis in a raptor handler and a wild bird fancier. Ann. Allergy. Asthma. Immunol. 74:437–441. 7749976

[10] Haitjema, T. , H. van Velzen‐Blad , and J. M. van den Bosch . 1992 Extrinsic allergic alveolitis caused by goose feathers in a duvet. Thorax 47:990–991. 1465764 10.1136/thx.47.11.990 PMC464133

[11] Kim, K. T. , J. W. Dalton , and W. B. Klaustermeyer . 1993 Subacute hypersensitivity pneumonitis to feathers presenting with weight loss and dyspnea. Ann. Allergy 71:19–23. 8328707

[12] Inase, N. , H. Sakashita , Y. Ohtani , Y. Sogou , Y. Sumi , T. Umino , Y. Usui , and Y. Yoshizawa . 2004 Chronic bird fancier's lung presenting with acute exacerbation due to use of a feather duvet. Intern. Med. 43:835–837. 15497520 10.2169/internalmedicine.43.835

[13] Craig, T. J. , J. Hershey , R. J. Engler , W. Davis , G. B. Carpenter , and K. Salata . 1992 Bird antigen persistence in the home environment after removal of the bird. Ann. Allergy 69:510–512. 1471783

[14] Curtis, L. , B. S. Lee , D. Cai , I. Morozova , J. L. Fan , P. Scheff , V. Persky , C. Einoder , and S. Diblee . 2002 Pigeon allergens in indoor environments: a preliminary study. Allergy 57:627–631. 12100304 10.1034/j.1398-9995.2002.03405.x

[15] Kuramochi, J. , N. Inase , K. Takayama , Y. Miyazaki , and Y. Yoshizawa . 2010 Detection of indoor and outdoor avian antigen in management of bird‐related hypersensitivity pneumonitis. Allergol. Int. 59:223–228. 20414051 10.2332/allergolint.09-OA-0161

[16] Tsutsui, T. , Y. Miyazaki , J. Kuramochi , K. Uchida , Y. Eishi , and N. Inase . 2015 The amount of avian antigen in household dust predicts the prognosis of chronic bird‐related hypersensitivity pneumonitis. Ann. Am. Thorac. Soc. 12:1013–1021. 26010749 10.1513/AnnalsATS.201412-569OC

[17] Ohtani, Y. , S. Saiki , Y. Sumi , N. Inase , S. Miyake , U. Costabel , and Y. Yoshizawa . 2003 Clinical features of recurrent and insidious chronic bird fancier's lung. Ann. Allergy. Asthma. Immunol. 90:604–610. 12839317 10.1016/S1081-1206(10)61863-7

[18] Ohtani, Y. , K. Kojima , Y. Sumi , M. Sawada , N. Inase , S. Miyake , and Y. Yoshizawa . 2000 Inhalation provocation tests in chronic bird fancier's lung. Chest 118:1382–1389. 11083690 10.1378/chest.118.5.1382

[19] Ishizuka, M. , Y. Miyazaki , T. Tateishi , T. Tsutsui , K. Tsuchiya , and N. Inase . 2015 Validation of inhalation provocation test in chronic bird‐related hypersensitivity pneumonitis and new prediction score. Ann. Am. Thorac. Soc. 12:167–173. 25562381 10.1513/AnnalsATS.201408-350OC

[20] Hargreave, F. E. , J. Pepys , J. L. Longbottom , and D. G. Wraith . 1966 Bird breeder's (fancier's) lung. Lancet 1:445–449. 4159692 10.1016/s0140-6736(66)91454-1

[21] Masuo, M. , Y. Miyazaki , K. Suhara , M. Ishizuka , T. Fujie , and N. Inase . 2016 Factors associated with positive inhalation provocation test results in subjects suspected of having chronic bird‐related hypersensitivity pneumonitis. Respir. Investig. 54:454–461. 10.1016/j.resinv.2016.05.002 27886857

