# Supplementary material for: Environmental levels of avian antigen are relevant to the progression of chronic hypersensitivity pneumonitis during antigen avoidance
Source: Immun Inflamm Dis. 2017 Nov 22;6(1):154–62. doi: 10.1002/iid3.202 (PMC5818447; doi:10.1002/iid3.202)
Supplement: Supplementary file 1 — Table S1. The classification of the patients with chronic bird‐related hypersensitivity pneumonitis, idiopathic interstitial pneumonias or home‐related HP, and other respiratory diseases into the high and low‐level exposure groups in the Questionnaire Dust Study. [file IID3-6-154-s001.docx]

| Number of patients | High-level exposure group | Low-level exposure group |
| --- | --- | --- |
| Chronic bird-related HP | 5 | 11 |
| IIPs or home-related HP | 6 | 24 |
| Other respiratory diseases | 2 | 5 |

Supplemental Table 1. The classification of the patients with chronic bird-related hypersensitivity pneumonitis, idiopathic interstitial pneumonias or home-related HP, and other respiratory diseases ^a^ into the high and low-level exposure groups in the Questionnaire Dust Study.

^a^Other respiratory diseases included microscopic polyangiitis, sleep apnea syndrome, bronchial asthma, bacterial pneumonia, pleural mesothelioma and sarcoidosis.

HP: hypersensitivity pneumonitis, IIPs: idiopathic interstitial pneumonias
